# Efficacy and safety of dobutamine versus milrinone in cardiogenic shock: Systematic review and meta-analysis

**DOI:** 10.21542/gcsp.2026.5

**Published:** 2026-02-28

**Authors:** Arga Setyo Adji, Rizka Kusuma Wardahni, Isnia Maulidah, Diski Saisa, Angela Puspita, Antonius Dwi Saputra, Aufa Baraja, Anne Mantofa, Intan Komalasari

**Affiliations:** 1 Department of Cardiology, Faculty of Medicine, Universitas Hang Tuah Surabaya, Indonesia; 2 Faculty of Medicine, Hang Tuah University, Surabaya, Indonesia; 3 Faculty of Medicine, University of Indonesia, Jakarta, Indonesia; 4 Emergency Medicine Division, Department of Internal Medicine, Hang Tuah University, Surabaya, East Java, Indonesia; 5 Faculty of Medicine, University of Jember, Jember, Indonesia; 6 Cardiovascular Subspecialist Study Program, Faculty of Medicine, Universitas Airlangga, Surabaya, East Java, Indonesia

## Abstract

**Background:** Cardiogenic shock (CS) is a life-threatening condition requiring timely pharmacological support. Dobutamine and milrinone are commonly used inotropes, yet their comparative efficacy and safety in CS management remain uncertain. This systematic review and meta-analysis aims to evaluate the outcomes associated with dobutamine versus milrinone in CS patients.

**Methods:** A thorough literature search was conducted across databases including MEDLINE (via PubMed), CENTRAL (Cochrane Central Register of Controlled Trials), and Scopus, spanning publications up to February 2025. Randomized controlled trials and observational studies comparing dobutamine and milrinone in adult patients diagnosed with CS were incorporated. Statistical analyses were performed employing a random-effects model. Effect measures comprised odds ratios (OR) for binary outcomes and standardized mean differences (SMD) for continuous outcomes, accompanied by 95% confidence intervals (CI), with heterogeneity evaluated using *I*^2^ statistics.

**Results:** Dobutamine was associated with higher in-hospital mortality (OR 1.56, 95% CI 1.01–2.39; *I*^2^ = 93%; *p* = 0.04), which was non-significant in RCTs (OR 1.24, 95% CI 0.70–2.19; *I*^2^ = 0%; *p* = 0.46; moderate certainty) but significant in observational studies (OR 1.63, 95% CI 1.02–2.59; *I*^2^ = 94%; *p* = 0.04; very low certainty). Mortality was markedly increased in ICU settings (OR 2.85, 95% CI 1.42–5.69; *I*^2^ = 93%; *p* < 0.001; moderate certainty), whereas non-ICU settings showed no significant difference (OR 0.68, 95% CI 0.32–1.45; *I*^2^ = 84%; low certainty). Overall all-cause mortality was also higher with dobutamine (OR 1.54, 95% CI 1.07–2.21; *I*^2^ = 87%; *p* = 0.02), remaining non-significant in RCT data (OR 1.30, 95% CI 0.73–2.32; moderate certainty) but significant in observational data (OR 1.58, 95% CI 1.06–2.35; *I*^2^ = 89%; low certainty). No significant differences were observed in ICU length of stay (SMD −0.13, 95% CI −0.99 to 0.73; *I*^2^ = 93%) or hospital length of stay (SMD −0.69, 95% CI −4.49 to 3.11; *I*^2^ = 98%), and significant arrhythmias were comparable (OR 0.88, 95% CI 0.40–1.93; *I*^2^ = 76%). However, acute renal failure was significantly more frequent with dobutamine (OR 1.22, 95% CI 1.01–1.47; *I*^2^ = 0%; *p* = 0.03).

**Conclusion:** Dobutamine is associated with increased mortality particularly in ICU patients and a higher risk of acute renal failure compared with milrinone, with evidence certainty ranging from moderate to very low and substantial heterogeneity across mortality outcomes.

## Background

Cardiogenic shock (CS) is a critical condition in which the heart suddenly becomes unable to pump enough blood to meet the body’s demands. This results in low cardiac output, which manifests clinically, biochemically, and hemodynamically as end-organ hypoperfusion^1,2^. CS continues to pose significant challenges for clinicians due to its high mortality rate. In the United States, approximately 40,000 to 50,000 individuals experience CS annually, with a mortality rate reaching nearly 50%^3^. CS is characterized by inadequate tissue perfusion caused by heart dysfunction. This condition typically involves hypotension (systolic blood pressure < 90 mmHg or mean arterial pressure (MAP) < 65 mmHg), signs of cardiac dysfunction (cardiac index < 2.2 L/min/m^2^, echocardiographic evidence of left or right ventricular dysfunction), and clinical features such as cold extremities, altered mental status, pulmonary congestion, and organ hypoperfusion indicators like elevated serum creatinine, serum lactate > 2 mmol/L, or SvO2 < 50%^4,5^.

CS frequently arises as a complication of acute coronary syndrome (ACS), with the highest incidence occurring during hospitalization. Hospital mortality for CS is significant, with most deaths occurring within 30 to 60 days following diagnosis. Data from intensive coronary care units (ICCU) in the US and Canada reveal that myocardial infarction (MI) is the most common cause of CS. Additionally, 18% of cases result from ischemic cardiomyopathy without ACS, 28% from ischemic cardiomyopathy, and 17% from other cardiac conditions^2^. Other potential causes of CS include severe valvular heart disease, myocarditis, Takotsubo cardiomyopathy, decompensated pre-existing cardiomyopathy, uncontrolled tachyarrhythmias, pulmonary embolism, and other conditions^6^. The primary treatment for CS involves the use of vasopressors and inotropes, which are widely recommended for managing acute heart failure with reduced cardiac output and ST-elevation myocardial infarction complicated by CS^7–9^.

The treatment approach for CS is generally divided into two key objectives: identifying and addressing the underlying cause, and improving hemodynamic stability using vasoactive medications. Although mechanical circulatory support has advanced, no studies have demonstrated significant clinical improvement from these interventions alone^6,10^. Vasopressors and inotropic agents play a crucial role in maintaining blood pressure by enhancing cardiac output and systemic vascular resistance to ensure adequate organ perfusion^3^. Research has shown that norepinephrine is more effective than dopamine or dobutamine in managing CS patients. However, debate continues regarding the preferred inotropic agent between dobutamine and milrinone, with studies indicating that both drugs produce similar clinical outcomes. Despite their effectiveness, both dobutamine and milrinone may increase left ventricular afterload, which raises myocardial oxygen demand, potentially worsening myocardial infarction severity and increasing the risk of arrhythmias. Consequently, careful titration, close monitoring of vital signs, cardiac output, and organ perfusion are essential when using these drugs^11^.

A prior meta-analysis by Abdel-Razek et al. (2023) compared dobutamine and milrinone in cardiogenic shock, focusing on mortality, ICU/hospital length of stay, and clinically significant arrhythmias, and included only two randomized trials. Building on this evidence, our meta-analysis expands the scope through an updated, comprehensive search and evaluates not only efficacy (mortality and ICU/hospital stay) but also clinically relevant safety endpoints, notably acute kidney injury. Collectively, these advances provide a more complete view of the net clinical benefit and directly address key limitations of the prior synthesis. Given these considerations, further research is necessary to evaluate the efficacy, safety, and risk of arrhythmic events (such as atrial or ventricular fibrillation) associated with dobutamine and milrinone in the treatment of CS.

## Methods

This systematic review was conducted in accordance with the PRISMA (Preferred Reporting Items for Systematic Reviews and Meta-Analyses) guidelines to ensure transparency and methodological rigor. The review has been formally registered with the International Prospective Register of Systematic Reviews (PROSPERO) under the registration number (CRD420251020467). By adhering to the PRISMA protocol, the review maintains a structured approach, minimizing bias and promoting comprehensive reporting of the included studies and their outcomes^12^.

### Eligibility criteria

For this meta-analysis, we adhered strictly to predefined inclusion and exclusion criteria to ensure the selection of relevant and high-quality studies. Cardiogenic shock (CS) was defined based on specific clinical and hemodynamic criteria, including hypotension (systolic blood pressure < 90 mmHg or mean arterial pressure (MAP) < 65 mmHg), signs of cardiac dysfunction (cardiac index < 2.2 L/min/m^2^, or echocardiographic evidence of left or right ventricular dysfunction), and clinical manifestations such as cold extremities, altered mental status, pulmonary congestion, and organ hypoperfusion markers like elevated serum creatinine, serum lactate levels exceeding two mmol/L, or SvO2 below 50%.

To meet the inclusion criteria, studies had to involve patients with confirmed CS and compare treatments involving dobutamine and milrinone. Studies that did not focus on CS patients were excluded. Several key selection criteria were applied to ensure consistency and relevance. First, efficacy outcomes considered included all-cause in-hospital mortality, overall mortality, length of hospital stay, and duration of stay in the intensive care unit (ICU). Second, safety outcomes assessed included in-hospital arrhythmias, hypotensive episodes, and acute renal failure. Third, we only included specific study designs such as cohort studies, case-control studies, and randomized controlled trials (RCTs), with all eligible studies required to be published in English. Lastly, we excluded trials that failed to compare dobutamine to milrinone, or lacked appropriate outcome measures. This meticulous selection process ensured that our meta-analysis incorporated only the most relevant and reliable studies, improving the robustness and validity of our findings.

### Search strategy and selection of studies

A comprehensive search was conducted across multiple databases to identify relevant studies published up until February 2025. The databases searched included MEDLINE (via PubMed), CENTRAL (Cochrane Central Register of Controlled Trials), and Scopus. The search strategy utilized a combination of key terms such as “Cardiogenic Shock”, “Dobutamine”, “Milrinone”, “Inotropic Agents”, “Vasopressors”, and “Hemodynamic Support”. These terms were combined using Boolean operators “AND” and “OR” to ensure a broad yet targeted search strategy. To maximize coverage, the reference lists of identified articles were also reviewed to locate additional studies that were relevant to our research focus. This thorough search process aimed to gather all available evidence comparing the efficacy and safety of dobutamine and milrinone in patients with cardiogenic shock, ensuring the inclusion of the most up-to-date data available.

### Data extraction

Designated investigators (A.S.A., A.B., M.Y., and B.G.L.) carefully extracted relevant data from the identified eligible studies using a predefined data extraction form. The data collected included various study characteristics such as the author, publication year, study design, country of origin, group allocations (dobutamine vs. milrinone), gender distribution (%), mean age (in years), reported side effects, and follow-up duration. Additionally, participant demographics, details of the interventions (such as the type and dosage of dobutamine or milrinone), and outcome measures were systematically recorded. To ensure the accuracy and integrity of the data, a rigorous cross-checking process was employed. An independent investigator reviewed the extracted data to confirm its accuracy and completeness, minimizing the risk of errors or omissions. This thorough validation process guaranteed the reliability and robustness of the data for the subsequent analysis.

### Quality assessment

All the included studies underwent further assessment using Newcastle Ottawa Scale (NOS) for non-randomized study design which has three components: Patient selection (four points), group comparability (one point), and exposure determination (three points). The overall score varied from 0 (worst) to 8 (best). Cochrane risk-of-bias tool for randomized trials ver. 2 (RoB 2) was also used to assess risk of bias in randomized controlled trial (RCT) studies. All calculations and assessments based on five major domains are carried out automatically on the RoB2 tools^13,14^. Three independent investigators (A.S.A., A.B, and A.M) executed the quality assessment process. Should any discrepancies arise during this evaluation phase, the investigators would collaboratively resolve them.

### Outcome measure

The analysis evaluated several outcome measures, encompassing both angiographic and clinical endpoints. Efficacy outcomes were based on all-cause in-hospital mortality, overall mortality, length of hospital stay, and ICU stay duration. Safety outcomes included the occurrence of in-hospital arrhythmias, and acute renal failure.

### Data synthesis and statistical analysis

Each outcome measure in this study was analyzed with its own pooled odds ratio (OR) and 95% confidence interval (CI), which were calculated using a meta-analysis. The *I*^2^ statistic was employed to assess the heterogeneity among the included studies. Patients with cardiogenic shock undergoing treatment with either dobutamine or milrinone were further categorized into subgroups based on acute decompensated heart failure-related cardiogenic shock (ADHF-CS) and acute myocardial infarction (AMI-CS). To ensure the robustness of the findings, a sensitivity analysis was also performed. Statistical significance was defined as a *p*-value of less than 0.05. All statistical analyses were conducted using Review Manager 5.4^15^.

## Results

### Study selection process and quality assessment

**Figure 1.**
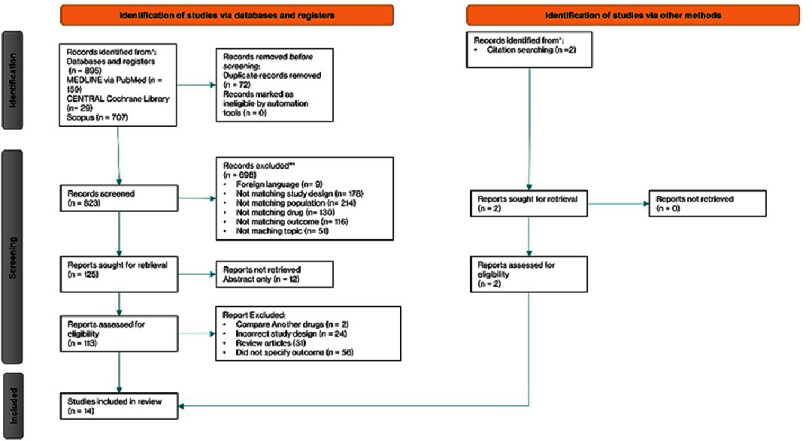
PRISMA flow diagram of the study selection process.

After conducting a comprehensive search across three major databases—MEDLINE (via PubMed), CENTRAL (Cochrane Central Register of Controlled Trials), and Scopus —a total of 895 records were identified. An additional two records were identified through other methods such as website searches and citation tracking. Before screening, 72 duplicate or otherwise ineligible records were removed (including those excluded by automation tools and other reasons), leaving 823 records for screening. Out of these, 698 records were excluded based on criteria such as foreign language (9 records), unmatched study design (178), population (214), drug (130), outcome (116), and topic (51). A total of 125 reports were sought for retrieval, but 12 were not retrieved due to being abstract-only. Among the 113 reports assessed for eligibility, 99 were excluded—2 for comparing different drugs, 24 due to incorrect study design, 31 due to review article and 56 for not specifying outcomes. In total, 14 studies met the inclusion criteria for the review. The full selection process is illustrated in the PRISMA flowchart (Figure 1).

### Study characteristics

Table 1 summarizes the outcomes of interest derived from a total of fourteen investigations, comprising 12 cohort studies and 2 randomized controlled trials (RCTs), involving a collective patient population of 23,559 individuals experiencing cardiogenic shock ^16–29^. The geographic distribution of these studies included ten conducted in the United States, three in Canada, and one in the Netherlands. Follow-up durations across these investigations ranged from twelve months to four years.

**Table 1:** Study characteristics.

No.	Author, Year	Design studies	Countries	Groups		Mean age (years)	Man, n (%)	Follow up (month)
				Dobutamine	Milrinone			
**1**	Abraham, 2005	Prospective Cohort	United States	4226	2021	70.4 ± 13.5 for Dobutamine and 67.3 ± 14.0 for Milrinone	4020 (64.4%)	During hospitalization
**2**	Aranda, 2003	RCT	United States	17	19	54 ± 9 for Dobutamine and 61 ± 8 for Milrinone	27 (75%)	During hospitalization
**3**	Arnold, 2006	Retrospective Cohort	United States	1311	433	63.1 ± 14.9 for Dobutamine and 61.0 ± 14 for Milrinone	1136 (65.1%)	During hospitalization
**4**	Berg, 2023	Retrospective Cohort	Netherlands	503	267	67.4 ± 12.1 for Dobutamine and 65.6 ± 12.1 for Milrinone	535 (69.5%)	Hospital discharge or in-hospital death
**5**	Gao, 2021	Retrospectice Cohort	United States	558	261	67.3 ± 14.5 for Dobutamine and 64.8 ± 13.2 for Milrinone	507 (61.9%)	Hospital discharge or in-hospital death
**6**	Hauptman, 2008	Retrospective Cohort	United States	8762	1949	N.A	N.A	Hospital discharge or in-hospital death
**7**	Lewis, 2019	Retrospective Cohort	United States	50	50	75 for Dobutamine and 72.5 for Milrinone	51 (51%)	Hospital discharge or in-hospital death
**8**	Mathew, 2021	RCT	Canada	96	96	72.0 ± 11.3 for Dobutamine and 68.9 ± 13.8 for Milrinone	122 (63.5%)	In-hospital death
**9**	Nandkeolyar, 2021	Retrospective Cohort	United States	256	70	N.A	N.A	In-hospital death
**10**	Rodenas-Alesina, 2023	Retrospective Cohort	Canada	207	366	63 (50-71) for Dobutamine and 60 (45-68) for Milrinone	406 (70.9%)	Hospital discharge or in-hospital death
**11**	Santo, 2023	Post hoc RCT	Canada	181	184	N.A	N.A	During hospitalization
**12**	Sasmita, 2023	Retrospective Cohort	United States	661	400	N.A	N.A	During hospitalization (30-days)
**13**	Scroggins, 2005	Retrospective Cohort	United States	40	27	N.A	N.A	Hospital discharge or in-hospital death
**14**	Yamani, 2001	Retrospective Cohort	United States	269	60	61 ± 11 for Dobutamine and 62 ± 12 for Milrinone	249 (75.7%)	Hospital discharge or in-hospital death

### Risk of bias

Table 2 presents the classification of the fourteen included studies based on the methodologies used to assess their risk of bias. Both randomized controlled trials (RCTs) were determined to have a low risk of bias according to the Cochrane Risk of Bias (RoB) assessment, indicating high-quality evidence^14^. The twelve cohort studies were assessed using the Newcastle–Ottawa Scale (NOS). Of these, six studies were rated at 7 out of 8 points (low risk of bias), three studies at 8 out of 8 points (low risk of bias), and three studies at 6 out of 8 points (moderate risk of bias)^13^. Overall, most studies demonstrated a low risk of bias, suggesting good methodological quality across the included research.

**Table 2:** Study outcomes.

**No.**	**Author, year**	**Drug, Comparator**	**Main result (Better> Worse)**	**Key Outcomes**	**Risk of bias/quality of study**	
**1**	Aranda, 2003 (RCT)	Dobutamine, Milrinone	Therapy lasted 50 ± 46 days for those in the dobutamine group and 63 ± 45 days in the milrinone group (*p* = .38). Pretransplantation mortality rate 1 patient on milrinone had cardiac arrest due to myocardial infarction (*p* = .53). The incidence of arrhythmia in both groups was the same, 9 patients (dobutamine vs milrinone; 52% *vs* 47%) and showed no significant difference (*P* = .5).	No difference in death, need additional vasodilator/inotropic therapy, mechanical cardiac support, between 2 group. The incidence of arrhythmia in both groups did not show any significant difference.	Under table	
**2**	Lewis, 2019 (Cohort)	Dobutamine, Milrinone	Length of ICU stay were similar between dobutamine (6 days) and milrinone (5 days) (*P* = .62). Length of hospital stay were similar too, dobutamine (12 days) and milrinone (11 days) (*P* = .39). For in-hospital mortality, there was no difference between dobutamine (10%) and milrinone (5%) (*p* = .20). The rate of arrhythmia was significantly higher in those receiving dobutamine compared to milrinone (62.9% vs 32.8%, *P*¡.01). The difference in arrhythmia was primarily driven by a significant difference in sinus tachycardia (Dobutamine vs Milrinone, 38.5% vs 25%, *P* = .02). The incidence of AF did not differ between the groups (Dobutamine vs Milrinone, 38.5% vs 60%, *P* = .55).	Length of ICU and hospital stay were similar between the groups. There was no difference observed in inhospital mortality. Arrhythmias were more common in patients treated with dobutamine than milrinone. The difference was primarily driven by a significant difference in sinus tachycardia and AF did not differ between the groups.	(7/8)	
**3**	Mathew, 2021 (RCT)	Dobutamine milrinone	In-hospital death from any cause occurred in 35 participants (37%) in the milrinone group and in 41 participants (43%) in the dobutamine group (RR, 0.85; 95% CI, 0.60 to 1.21). There were no significant differences between the groups in total hospital length of stay (Dobutamine vs Milrinone, IQR 4.5 day vs 5.5 day), or ICU length of stay (Dobutamine vs Milrinone, IQR 15 day vs 16 day)	There were no significant differences between the groups in-hospital death, resuscitated cardiac arrest, total hospital LOS, or ICU LOS	Under table	
**4**	Yamani, 2001 (Cohort)	Dobutamine milrinone	This study showed no difference in in-hospital mortality between the dobutamine group (7.8%) and the milrinone group (10%) (*P* = 0.60). The incidence of ventricular arrhythmias also did not show significant results, both in sustained ventricular tachycardia (dobutamine vs milrinone, 3% vs 5%, *P* = 0.46) and non-sustained tachycardia (dobutamine vs milrinone, 24% vs 15%, *P* = 0.12). The average LOS in hospital between dobutamine (3.3 days) and milrinone (3.2 days) was not much different (*P* = 0.64)	There was no significant difference in the incidence of ventricular arrhythmias. Likewise, the in-hospital mortality rate was similar. Both dobutamine and milrinone groups had similar hospital lengths of stay	(7/8)	
**5**	Gao, 2021 (Cohort)	Inotropes, non-inotropes	Among patients receiving inotrope agents, those receiving dobutamine (HR: 0.882, 95% CI: 0.820–0.949, *p*<0.001) had lower risk of ICU mortality, but significantly increased the risk of hospital mortality (HR: 1.758, 95% CI 1.467–2.108, *p*<0.001). In contrast, milrinone (HR: 0.559, 95% CI: 0.430–0.727, *p*<0.001) significantly decreased the risk of hospital mortality. Milrinone had no risk of ICU mortality (HR 1.068, 95% CI 0.964, 1.184; *P* = 0.208)	Dobutamine had lower risk ICU mortality, but significantly increased the risk of hospital mortality. Milrinone significantly decreased the risk of hospital mortality, but no effect on ICU mortality	(6/8)	
**6**	Arnold, 2006 (Cohort)	Nesiritide, dobutamine, milrinone	With nesiritide, the in-hospital mortality rate (2.9%) was lower than with dobutamine (10.2%) or milrinone (7.9%)(*p*<0.001). The study observed hospital LOS for patients treated with nesiritide (7.0 ± 5.3 days, median 5 days) was significantly lower than that observed in those treated with dobutamine (10.4 ± 12.9 days, median 7 days) and milrinone (12.2 ± 29.9 days, median 7 days) (*p*<0.001). The average ICU LOS for nesiritide (1.1 ± 2.9) was lower than for dobutamine (3.4 ± 9.8) and milrinone (3.9 ± 6.8)	Hospital mortality rate of patient treated with nesiritide was lower than dobutamine or milrinone. LOS in ICU and hospital was lower in nesiritide group compared to dobutamine dan milrinone group.	(7/8)	
**7**	Berg, 2023 (Cohort)	Dobutamine and milrinone	30-day mortality was significantly lower in the dobutamine group rather than milrinone group (41.7% vs. 50.8%, unadjusted *p* = 0.020)	30-day mortality was lower in the dobutamine group than in the milrinone group.	(8/8)	
**8**	Hauptman, 2008 (Cohort)	Dobutamine and milrinone	Mortality rate in the dobutamine group was 7.8% of 8762 cases, while in the milrinone group was 7.1% of 1949 cases	There was no significant difference in mortality rates between the two groups.	(8/8)	
**9**	Nandkeolyar, 2021 (Cohort)	Dobutamine and milrinone	The mortality rate with dobutamine alone was 15% (*n* = 137) and milrinone alone was 3% (*n* = 35). 15% increased risk of mortality are found per each 1 μg/kg/minute increase in dobutamine, and dose > 3 μg/kg/min are associated with 3-fold increased risk of mortality in CS.	Dobutamine was associated with higher mortality overall.	(7/8)	
**10**	Rodenas, 2023 (Cohort	Dobutamine and milrinone	During the first 30 days following CICU admission, there were 133 deaths: 76 (36.7%) in the dobutamine group and57 (15.6%) in the milrinone group. Those receiving milrinone had lower rates of mortality (unadjusted HR = 0.36, 95% CI0.25–0.52). The average LOS (length of stay) in the CICU of both groups was almost the same, 6 day (p = .963), while the average LOS in the hospital in the miorinone group was longer (23 days) compared to the dobutamine group (17 days) p < .001)	Mortality in the first 30 days in the dobutamine group was higher than in the milrinone group. There was no significant difference in the CICU LOS of the two groups, but the hospital LOS was significantly longer in the milrinone group than dobutamine group	(7/8)	
**11**	Santo, 2023 (Cohort)	Dobutamine and milrinone	In the group without AKI, the use of milrinone was associated with a reduced risk of the primary outcome (RR: 0.48; 95% CI 0.24–0.97; *P* = 0.04). Conversely, in the group with AKI, the risk of the primary outcome was similar between milrinone and dobutamine (RR: 1.06; 95% CI 0.78–1.46; *P* = 0.70)	In patients without AKI, milrinone use was linked to lower rates of the primary outcome and mortality compared to dobutamine	(6/8)	
**12**	Sasmita, 2023 (Cohort)	Dobutamine and milrinone	HR for all-cause mortality of dobutamine group was 1.149 (95%CI 1.002–1.319), while milrinone was 0.547 (95%CI 0.449–0.666)	Dobutamine was associated with a higher mortality risk, while milrinone was associated with a reduction of 30-day all-cause mortality	(7/8)	
**13**	Scroggins, 2005 (Cohort)	Dobutamine and milrinone	In the dobutamine group, the mean LOS hospital was 9.6 days while the LOS ICU was 3.5 days. In the milrinone group, the mean LOS hospital was 8.96 days, while the ICU LOS was 2.70 days. The in-hospital mortality rate was 5% in the dobutamine group, 18% in the milrinone group, and 6% in the nesiritide group patients	Hospital LOS and ICU LOS of the dobutamine group were longer than the milrinone group. The mortality rate of dobutamine group was lower than milrinone group.	(7/8)	
**14**	Abraham, 2005 (Cohort)	Dobutamine and milrinone	The average ICU length of stay data showed that the duration of each group was not much different (milrinone vs dobutamine; 6.9 days vs 6.1 days). Likewise, the average hospital length of stay showed a duration that was not much different (milrinone vs dobutamine; 10.9 days vs 10 days). The percentage of mortality for milrinone group was lower than the dobutamine group (milrinone vs dobutamine; 12.3% vs 13.9%). OR for mortality of dobutamine group compared to milrinone group after adjusted for covariates and propensity score was 1.24 (95% CI 1.03 to 1.55, *p* = 0.027)	ICU length of stay and hospital length of stay in both groups did not differ much. However, the mortality rate of dobutamine group was significantly higher than the milrinone group.	(6/8)	

**Notes.**

Abbreviations AF atrial fibrillation AKI acute kidney injury CI confidence interval CICU cardiac intensive care unit CS cardiogenic shock ICU intensive care unit IQR interquartile range LOS length of stay OR odds ratio

### Efficacy of dobutamine versus milrinone

**Figure 2.**
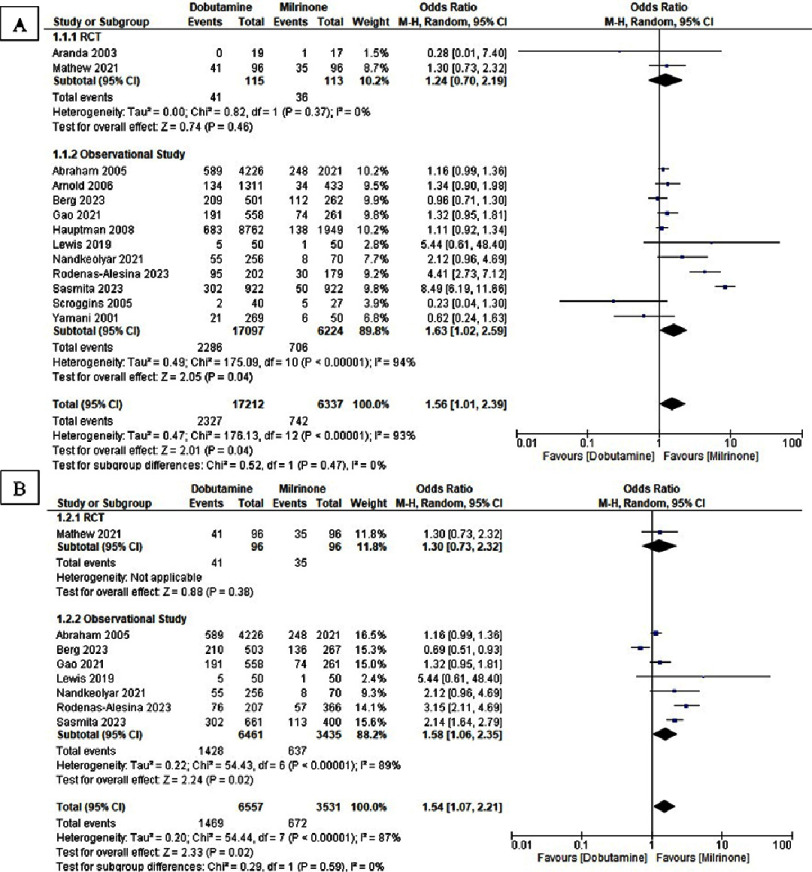
Forest plot of dobutamine vs milrinone of (A) in-hospital mortality and (B) all-cause mortality.

**Figure 3.**
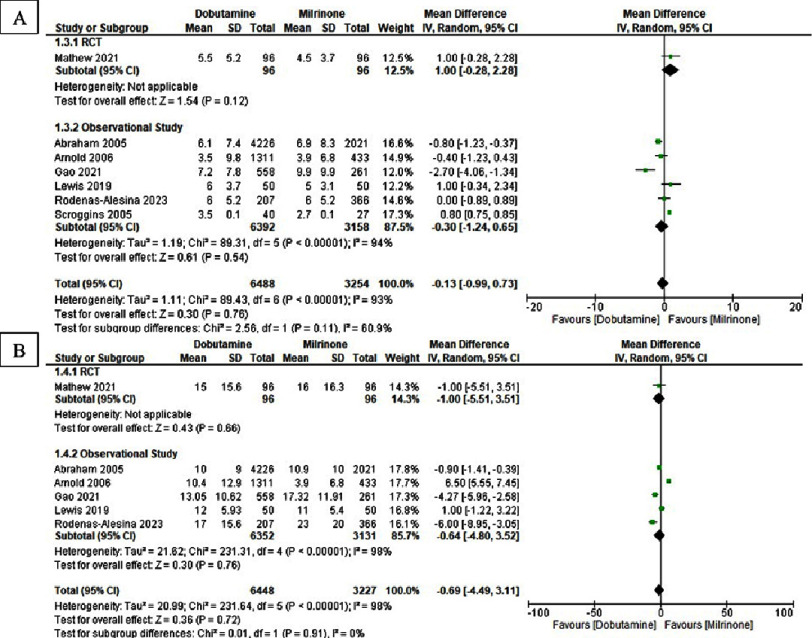
Forest plot of dobutamine vs milrinone in (A) length-of stay in ICU and (B) length-of stay in hospital.

All-cause in-hospital mortality and overall all-cause mortality were assessed (Figure 2), while ICU and total hospital length of stay are shown in Figure 3. An increased risk of all-cause in-hospital mortality was observed with dobutamine compared with milrinone (OR 1.56, 95% CI: 1.01–2.39; *I*^2^ = 93%; *p* = 0.04), alongside a similarly elevated overall all-cause mortality (OR 1.54, 95% CI: 1.07–2.21; *I*^2^ = 87%; *p* = 0.02). No statistically significant difference was identified in ICU length of stay (SMD −0.13, 95% CI: −0.99 to 0.73; *I*^2^ = 93%; *p* = 0.76) or total hospital stay (SMD −0.69, 95% CI: −4.49 to 3.11; *I*^2^ = 98%; *p* = 0.72).

**Figure 4.**
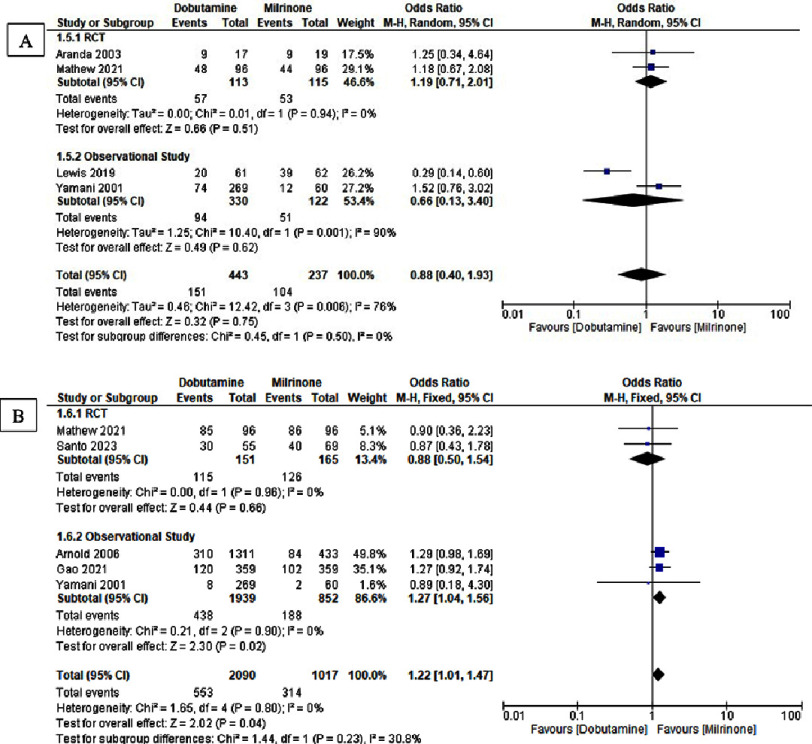
Forest plot of dobutamine vs milrinone in (A) significant arrhythmias in hospitalized patient and (B) acute renal failure.

**Figure 5.**
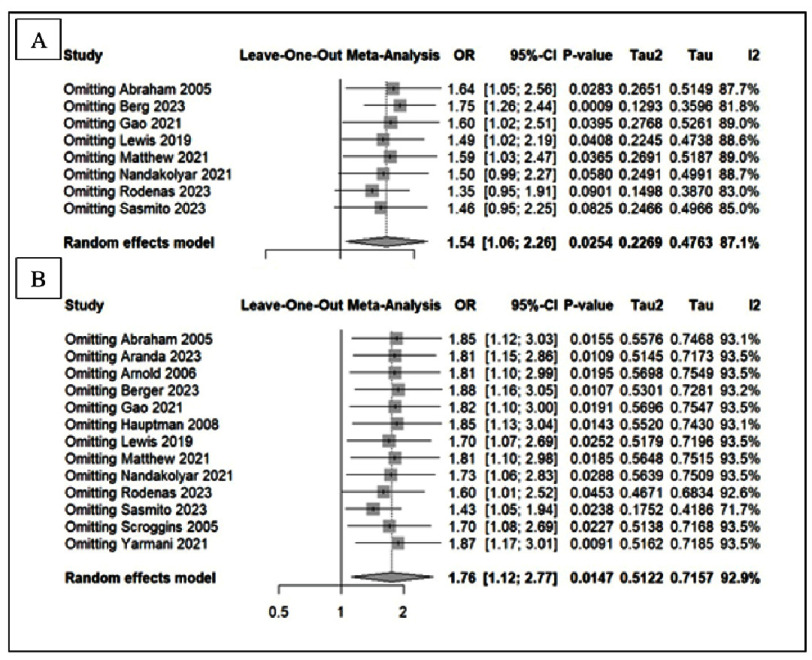
Sensitivity analysis of dobutamine vs milrinone in (A) all-cause mortality, (B) in hospital mortality.

Sensitivity analyses using a leave-one-out approach for length-of-stay outcomes are shown in Figure 5. For ICU stay, the pooled estimate remained essentially unchanged (MD −0.13, 95% CI −1.02 to 0.75), and exclusion of individual studies produced only small variations, with mean differences ranging approximately from −0.32 to 0.18. Heterogeneity remained high across all iterations, with *I*^2^ values ranging from 78.8% to 94.4%. A similar pattern was observed for total hospital stay, where the overall effect remained non-significant (MD −0.18, 95% CI −3.39 to 3.03), and individual exclusions yielded mean differences between −1.66 and 0.70. Despite these fluctuations, *I*^2^ remained consistently elevated (approximately 97–98%), indicating that the variability is not attributable to a single influential study.

**Figure 6.**
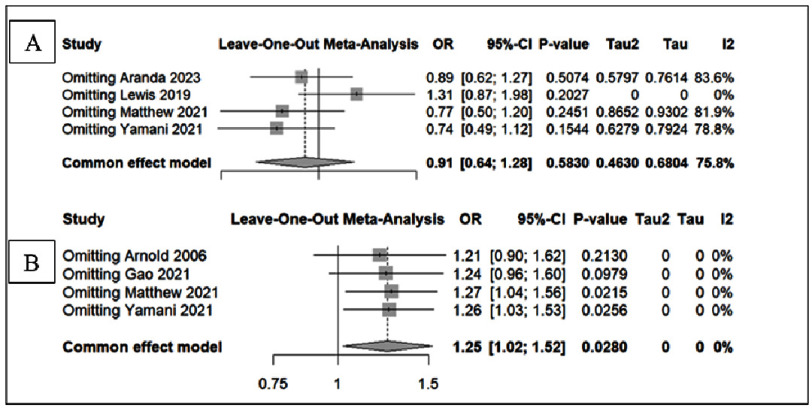
Sensitivity analysis of dobutamine vs milrinone in (A) significant arrhythmias in hospitalized patient and (B) acute renal failure.

For mortality outcomes (Figure 6), the leave-one-out analyses likewise showed stable results. In overall all-cause mortality, the pooled effect remained significant (OR 1.54, 95% CI 1.06–2.26; *I*^2^ = 87.1%), with individual exclusions producing ORs ranging from 1.35 to 1.75. For in-hospital mortality, the pooled estimate was 1.76 (95% CI 1.12–2.77; *I*^2^ = 92.9%), and omission of individual studies yielded ORs between 1.43 and 1.88. Although heterogeneity persisted (*I*^2^ generally above 90% for in-hospital mortality), no single study altered the direction of effect or rendered the association non-significant, supporting the overall consistency of the mortality findings.

In Figure S3 (subgroup based on setting), the pooled analysis shows a clear difference between ICU and non-ICU populations. In the ICU subgroup, the random-effects model yielded an OR of 2.85 (95% CI 1.42–5.69) with substantial heterogeneity (*I*^2^ 93.6%, *p* < 0.0001), indicating a significantly higher odds of the outcome with milrinone compared to dobutamine in critically ill patients treated in intensive care. In contrast, the non-ICU subgroup showed an OR of 0.68 (95% CI [0.32–1.45]) with *I*^2^ 84.3%, which was not statistically significant.

The test for subgroup differences was significant under the random-effects model (*p* = 0.0061), suggesting that treatment setting contributes meaningfully to between-study heterogeneity. In Figure S4 (subgroup based on mechanical circulatory support), neither the No-MCS group (OR 0.58 [0.31–1.08], *I*^2^ 92.5%) nor the Mixed/MCS group (OR 0.65 [0.36–1.16], *I*^2^ 91.0%) demonstrated a statistically significant effect under the random-effects model. The interaction test between subgroups was not significant (*p* = 0.1229), indicating that the use of mechanical circulatory support did not significantly modify the treatment effect.

In Figure S5 (subgroup based on adjustment method), results varied according to analytical approach. Unadjusted studies showed a significant pooled effect (OR 1.47 [1.12–1.92], *I*^2^ 0%), whereas studies using propensity score adjustment had high heterogeneity (*I*^2^ 95.2%) and a non-significant pooled estimate under random effects (OR 0.53 [0.20–1.42]). The multivariable regression study (OR 0.75 [0.51–1.11]) and the randomized controlled trial (OR 0.81 [0.46–1.43]) were also not statistically significant. The subgroup difference was significant under the random-effects model (*p* = 0.0101), indicating that the statistical adjustment method influenced the estimated treatment effect. In Figure S6 (subgroup based on underlying disease), the ADHF-CS subgroup showed a random-effects OR of 0.59 (95% CI 0.35–1.00; *I*^2^ 88.7%), while the AMI-CS subgroup showed an OR of 0.69 (95% CI 0.10–4.82; *I*^2^ 70.3%). The interaction test was not significant (*p* = 0.8736), suggesting no clear evidence that the underlying cause of cardiogenic shock altered the comparative effect of the two inotropes.

### Safety of dobutamine versus milrinone

The comparison of dobutamine and milrinone regarding significant arrhythmias and acute renal failure is presented in Figure 4. No significant difference was observed in the incidence of significant arrhythmias (OR 0.88, 95% CI: 0.40–1.93; *I*^2^ = 76%; *p* = 0.75). However, dobutamine was associated with a higher risk of acute renal failure compared with milrinone (OR 1.22, 95% CI 1.01–1.47; *I*^2^ = 0%; *p* = 0.03). The detailed results are summarized in Table 3.

**Table 3:** Summary of results and certainty assessment.

End point	No. Studies	Total Population (n)	Effect Size [95% CI]	Heterogeneity	*p* value	Certainty (GRADE)
Efficacy						
**All-cause in-hospital mortality**	13	23549	OR 1.56 [1.01–2.39]	93%	0.04^*^	
RCT	2	228	OR 1.24 [0.70–2.19]	0%	0.46	⨁⨁○○ Low^a^^,^^b^^,^^c^^,^^d^
Observational Study	11	23321	OR 1.63 [1.02–2.59]	94%	0.04	⨁○○○ Very low^a^^,^^c^^,^^e^
Subgroup (based on underlying disease)						
**ADHF-CS**	**5**	**8,274**	**OR 0.86 [0.55–1.36]**	**92%**	**0.53**	
**AMI-CS**	**2**	**870**	**OR 0.79 [0.32–1.96]**	**88%**	**0.61**	
Subgroup (based on adjustment method )						
**Unadjusted**	**4**	**1266**	**OR 1.47 [1.12–1.92]**	**0%**	**0.66**	
**Propensity Score**	**2**	**6820**	**OR 0.53 [0.20–1.42]**	**95%**	< 0.001*	
**Multivariate regression**	**1**	**1744**	**OR 0.75 [0.51–1.11]**	**NA**	**NA**	
**RCT**	**1**	**192**	**OR 0.81 [0.46–1.43]**	**NA**	**NA**	
Subgroup (based on setting)						
**ICU**	**6**	**3662**	**OR 2.85 [1.42–5.69]**	**93%**	< 0.001*	
**Non-ICU**	**6**	**19134**	**OR 0.68 [0.32–1.45]**	**84%**	**< 0.001**	
Subgroup (based on mechanical circulatory support)						
**MCS**	**5**	**1738**	**OR 0.58 [0.31–1.08]**	**92%**	< 0.0001*	
**No-MCS**	**1**	**100**	**OR 0.65 [0.36–1.16]**	**91%**	**0.10**	
**All-cause mortality**	8	10088	OR 1.54 [1.07–2.21]	87%	0.02^*^	
RCT	1	192	OR 1.30 [0.73–2.32]	NA.	0.38	⨁⨁⨁○ Moderate^c^^,^^d^^,^^f^
Observational Study	7	9896	OR 1.58 [1.06–2.35]	89%	0.02	⨁⨁○○ Low^a^^,^^c^^,^^d^^,^^g^
**ICU length of stay**	6	9742	SMD −0.13 [−0.99, 0.73]	93%	0.76	
RCT	1	192	MD 1.00 [−0.28–2.28]	Not applicable	0.12	⨁⨁⨁○ Moderate^c^^,^^d^^,^^f^
Observational Study	5	9550	MD −0.30 [−1.24–0.65]	94%	0.54	⨁○○○ Very low^a^^,^^b^^,^^c^^,^^e^
**Hospital length of stay**	6	9675	SMD −0.69 [−4.49, 3.11]	98%	0.72	
RCT	1	192	MD −1.00 [−5.51–3.51]	Not applicable	0.66	⨁⨁⨁○Moderate^c^^,^^d^^,^^f^
Observational Study	5	9483	MD −0.64 [−4.80–3.52]	98%	0.76	⨁○○○Very low^a^^,^^b^^,^^c^^,^^e^
Safety						
**Significant arrhythmias**	4	680	OR 0.88 [0.40–1.93]	76%	0.75	
RCT	2	228	OR 1.19 [0.71–2.01]	0%	0.51	⨁⨁⨁○Moderate^a^^,^^b^^,^^d^
Observational Study	2	452	OR 0.66 [0.13–3.40]	90%	0.62	⨁○○○Very low^b^^,^^c^^,^^d^^,^^e^
**Acute renal failure**	5	3107	OR 1.22 [1.01–1.47]	0%	0.03^*^	
RCT	2	316	OR 0.88 [0.50–1.54]	0%	0.66	⨁⨁⨁○Moderat^e^^,^^c^^,^^d^
Observational Study	3	2791	OR 1.27 [1.04–1.56]	0%	0.02	⨁⨁⨁⨁High

**Notes.**

Abbreviations: CI confidence interval ICU intensive care unit IQR interquartile range OR ordinal ratio SMD standardized mean difference NA not applicable

a Risk of bias are unlikely to lower confidence in the estimate of effect.

b There is variation in direction of effect between studies.

c Wide confidence intervals.

d Number of events does not meet the optimal information size.

e Statistical analyses showing considerable heterogeneity.

f None as only a single study was included for this outcome.

g Statistical analyses showing substantial heterogeneity.

**Figure 7.**
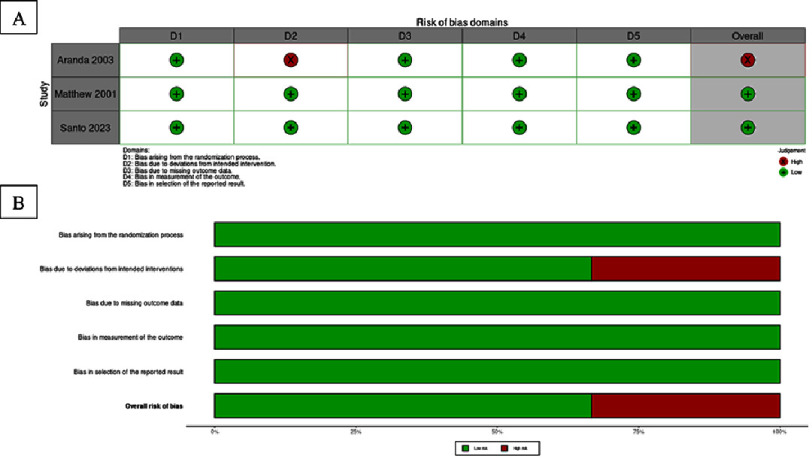
Risk of Bias Study of RCTs. (A) detailed risk of bias for each domain, (B) overall risk of bias.

Leave-one-out sensitivity analyses for significant arrhythmias are presented in Figure 7A. When individual studies were removed sequentially, the pooled odds ratios ranged from 0.74 to 1.31. Specifically, omission of Aranda 2023 yielded an OR of 0.89 (95% CI 0.62–1.27; *I*^2^ = 83.6%), while exclusion of Lewis 2019 resulted in an OR of 1.31 (95% CI 0.87–1.98; *I*^2^  =  0%). Removing Matthew 2021 produced an OR of 0.77 (95% CI 0.50–1.20; *I*^2^ = 81.9%), and exclusion of Yamani 2021 yielded an OR of 0.74 (95% CI 0.49–1.12; *I*^2^ = 78.8%). Despite some variation in heterogeneity, none of these analyses altered the overall non-significant finding (random-effects OR 0.91, 95% CI 0.64–1.28; *I*^2^ = 75.8%), indicating that no single study drove the arrhythmia result.

For acute renal failure (Figure 7B), the sensitivity analysis showed consistent findings across all iterations. The pooled estimate remained significant, with odds ratios ranging between 1.21 and 1.27 depending on the omitted study. For example, exclusion of Arnold 2006 resulted in an OR of 1.21 (95% CI 0.99–1.61), while omission of Gao 2021 yielded an OR of 1.24 (95% CI 0.96–1.60). Excluding Matthew 2021 and Yamani 2021 produced ORs of 1.27 (95% CI 1.04–1.63) and 1.26 (95% CI 1.03–1.53), respectively. Importantly, heterogeneity remained absent throughout (*I*^2^  =  0%), and the overall pooled estimate was stable (OR 1.25, 95% CI 1.02–1.52; *p* = 0.028), supporting the robustness of the association between dobutamine and acute renal failure.

## Discussion

Our systematic review and meta-analysis evaluating the safety and efficacy of dobutamine versus milrinone in cardiogenic shock demonstrated significant differences between the two inotropes.

Compared with milrinone, dobutamine was associated with higher all-cause in-hospital mortality (OR 1.56, 95% CI: 1.01–2.39; *I*^2^ = 93%; *p* = 0.04) and increased overall all-cause mortality (OR 1.54, 95% CI: 1.07–2.21; *I*^2^ = 87%; *p* = 0.02). These findings are consistent with prior evidence, including the meta-analysis by Biswas et al., which reported a relative risk of 0.87 (95% CI: 0.79–0.97; *p* < 0.05) favoring milrinone in acute decompensated heart failure.

The pharmacological profile of milrinone may partly explain this observed mortality benefit, as its phosphodiesterase-3 inhibition produces inotropic and vasodilatory effects that enhance cardiac output without substantially increasing myocardial oxygen demand, potentially reducing ischemic burden compared with *β*-adrenergic stimulation from dobutamine^30,31^. Conversely, the *β*-adrenergic stimulation of dobutamine might raise myocardial oxygen demand, thereby exacerbating myocardial ischemia and maybe leading deleterious consequences.

Dobutamine was not associated with a longer ICU stay compared with milrinone (SMD −0.13, 95% CI: −0.99 to 0.73; *I*^2^ = 93%; *p* = 0.76). Although prolonged ICU stay is clinically relevant due to increased costs and risk of nosocomial complications, no significant difference was observed between the two agents.

Regarding safety outcomes, there was no significant difference in the incidence of significant arrhythmias (OR 0.88, 95% CI: [0.40–1.93]; *I*^2^ = 76%; *p* = 0.75), consistent with the findings of the DOREMI trial, which reported comparable arrhythmic events between inotropes. However, dobutamine was associated with a higher risk of acute renal failure (OR 1.22, 95% CI: 1.01–1.47; *I*^2^ = 0%; *p* = 0.03), suggesting a potential renal advantage with milrinone, possibly related to its vasodilatory properties and more favorable hemodynamic profile^18,20^.

Dobutamine, a commonly used *β*-adrenergic agonist that augments cardiac output, was associated with higher mortality compared with milrinone. The pooled analysis demonstrated increased all-cause in-hospital mortality (OR 1.56, 95% CI: [1.01–2.39]; *I*^2^ = 93%; *p* = 0.04) and higher overall all-cause mortality (OR 1.54, 95% CI: 1.07–2.21; *I*^2^ = 87%; *p* = 0.02) with dobutamine. The *β*_1_-adrenergic stimulation produced by dobutamine may increase myocardial oxygen consumption and predispose to tachyarrhythmias, potentially contributing to adverse cardiovascular outcomes.

In contrast, milrinone, a phosphodiesterase-3 inhibitor, enhances intracellular cAMP independently of *β*-adrenergic receptors, promoting inotropy and vasodilation without substantially increasing myocardial oxygen demand. Prior evidence, including the meta-analysis by Biswas et al., reported a relative risk of 0.87 (95% CI: 0.79–0.97; *p* < 0.05) favoring milrinone, particularly in patients with acute decompensated heart failure, supporting the observed mortality advantage^30^.

While dobutamine’s pro-arrhythmic effects increased mortality risks, research by Abdel-Razek et al. indicates that milrinone showed a tendency towards lower mortality, especially in those with low cardiac output conditions or cardiogenic shock. Moreover, a retrospective analysis by Nielsen et al. found that intraoperative milrinone usage in cardiac surgery was linked with reduced mortality than dobutamine, therefore underlining the advantage of milrinone’s balanced inotropic and vasodilatory effects^31,32^.

These results show that milrinone is preferred especially in individuals with cardiogenic shock or heart failure when the danger of arrhythmias from dobutamine may offset its hemodynamic advantages. Their different pharmacological profiles help to explain the purported mortality benefit of milrinone over dobutamine. More suitable for those currently on beta-blocker treatment, milrinone’s vasodilatory effects reduce afterload without significantly increasing heart rate. Furthermore, unlike dobutamine, which activates beta-1 receptors and may cause heart rate and arrhythmia problems, milrinone’s non-beta-adrenergic impact reduces the risk of tachyarrhythmias^33^.

The overall pooled analysis demonstrated that dobutamine was associated with a higher risk of mortality compared with milrinone for both in-hospital (OR 1.56, 95% CI 1.01–2.39; *I*^2^ = 93%; *p* = 0.04) and overall mortality (OR 1.54, 95% CI [1.07–2.21]; *I*^2^ = 87%; *p* = 0.02). Given the substantial heterogeneity, subgroup analyses were performed. In Figure S3 (subgroup by clinical setting), ICU populations showed significantly increased mortality with dobutamine under the random-effects model (OR 2.85, 95% CI 1.42–5.69; *I*^2^ = 93.6%; *p* < 0.0001), whereas the non-ICU subgroup did not demonstrate a statistically significant difference (OR 0.68, 95% CI 0.32–1.45; *I*^2^ = 84.3%). The test for subgroup interaction was significant (*p* = 0.0061), indicating that treatment setting contributed meaningfully to between-study heterogeneity. These findings align with differences observed across study designs: randomized evidence such as the DOREMI trial did not show a clear mortality difference, whereas large observational registry analyses, including Abraham 2005 in acute decompensated heart failure, suggested worse outcomes with dobutamine, potentially explaining the stronger signal observed in ICU-predominant cohorts ^18,29^.

In Figure S4 (subgroup based on mechanical circulatory support), neither the No-MCS subgroup (OR 0.58, 95% CI 0.31–1.08; *I*^2^ = 92.5%) nor the Mixed/MCS subgroup (OR 0.65, 95% CI [0.36–1.16]; *I*^2^ = 91.0%) reached statistical significance, and the interaction test was not significant (*p* = 0.1229), indicating no clear modifying effect of MCS status.

Figure S5 (subgroup based on adjustment method) demonstrated important differences according to analytical approach: unadjusted analyses showed a significant pooled effect (OR 1.47, 95% CI [1.12–1.92]; *I*^2^ = 0%), whereas propensity score–adjusted studies exhibited substantial heterogeneity (*I*^2^ = 95.2%) with a non-significant pooled estimate under random effects (OR 0.53, 95% CI 0.20–1.42). The subgroup interaction was statistically significant (*p* = 0.0101), highlighting the impact of statistical methodology on effect estimates.

In Figure S6 (subgroup based on underlying disease), the ADHF-CS subgroup showed a random-effects OR of 0.59 (95% CI 0.35–1.00; *I*^2^ = 88.7%), while the AMI-CS subgroup yielded an OR of 0.69 (95% CI 0.10–4.82; *I*^2^ = 70.3%), with no significant interaction (*p* = 0.8736). These findings suggest that underlying etiology and analytical adjustment strategies substantially influence comparative effectiveness estimates, consistent with prior observational data demonstrating outcome variability according to clinical context and patient severity^17^.

### Limitations of current study

The present review has several important limitations. First, substantial between-study heterogeneity particularly for ICU and hospital length of stay undermines the stability of pooled estimates, and was not systematically explored with sensitivity analyses (e.g., leave-one-out) or meta-regression due to limited studies. Second, the evidence base is dominated by observational studies with variable adjustment sets, alongside mixed effect measures (adjusted vs unadjusted; OR vs RR), which increases risk of confounding and complicates interpretability. For future research, high quality of RCTs of dobutamine vs milrinone with protocolized dosing and co-interventions; pre-specify subgroups by shock phenotype (AMI-CS vs ADHF-CS) are required.

The predominance of observational studies (12 of 14 included studies) raises concerns regarding confounding by indication. In clinical practice, the choice between dobutamine and milrinone is not randomized but influenced by patient severity, hemodynamic profile, and institutional preference. Although several included studies applied multivariable adjustments or propensity matching, residual confounding cannot be excluded.

## Conclusion

Milrinone is associated with lower in-hospital and overall mortality compared with dobutamine in patients with cardiogenic shock, while no significant differences were observed in ICU or total hospital length of stay. Although arrhythmic events were comparable between agents, dobutamine was linked to a higher risk of acute renal failure. These findings suggest that the potential hemodynamic benefits of dobutamine must be weighed against its association with increased mortality and renal complications. Given the substantial heterogeneity and the variability across study designs, treatment decisions should be individualized based on patient profile, clinical setting—particularly ICU status—and risk of organ dysfunction. Further high-quality randomized evidence is warranted to clarify the optimal inotropic strategy in cardiogenic shock.

## Funding

This research did not receive any specific grant from funding agencies in the public, commercial, or not-for-profit sectors.

## Abbreviations

ACS: Acute coronary syndrome
CI: Confidence interval
CS: Cardiogenic shock
GCS: Glasgow Coma Scale
ICCU: Intensive coronary care unit
ICU: Intensive care unit
*I* ^2^: I-squared statistic
MAP: Mean arterial pressure
MI: Myocardial infarction
OR: Odds ratio
PRISMA: Preferred Reporting Items for Systematic Reviews and Meta-Analyses
PROSPERO: International Prospective Register of Systematic Reviews
RCT: Randomized controlled trial
SMD: Standardized mean difference
SvO2: Mixed venous oxygen saturation
